# Mitochondrial respiratory dysfunction disturbs neuronal and cardiac lineage commitment of human iPSCs

**DOI:** 10.1038/cddis.2016.484

**Published:** 2017-01-12

**Authors:** Mutsumi Yokota, Hideyuki Hatakeyama, Yasuha Ono, Miyuki Kanazawa, Yu-ichi Goto

**Affiliations:** 1 Department of Mental Retardation and Birth Defect Research, National Institute of Neuroscience, National Center of Neurology and Psychiatry, Tokyo 187-8502, Japan; 2 AMED-CREST, Japan Agency for Medical Research and Development, Tokyo 100-0004, Japan; 3 Medical Genome Center, National Center of Neurology and Psychiatry, Tokyo 187-8551, Japan

## Abstract

Mitochondrial diseases are genetically heterogeneous and present a broad clinical spectrum among patients; in most cases, genetic determinants of mitochondrial diseases are heteroplasmic mitochondrial DNA (mtDNA) mutations. However, it is uncertain whether and how heteroplasmic mtDNA mutations affect particular cellular fate-determination processes, which are closely associated with the cell-type-specific pathophysiology of mitochondrial diseases. In this study, we established two isogenic induced pluripotent stem cell (iPSC) lines each carrying different proportions of a heteroplasmic m.3243A>G mutation from the same patient; one exhibited apparently normal and the other showed most likely impaired mitochondrial respiratory function. Low proportions of m.3243A>G exhibited no apparent molecular pathogenic influence on directed differentiation into neurons and cardiomyocytes, whereas high proportions of m.3243A>G showed both induced neuronal cell death and inhibited cardiac lineage commitment. Such neuronal and cardiac maturation defects were also confirmed using another patient-derived iPSC line carrying quite high proportion of m.3243A>G. In conclusion, mitochondrial respiratory dysfunction strongly inhibits maturation and survival of iPSC-derived neurons and cardiomyocytes; our presenting data also suggest that appropriate mitochondrial maturation actually contributes to cellular fate-determination processes during development.

Mitochondria possess multiple copies of their own genome (mitochondrial DNA; mtDNA) and play some crucial roles in cellular energy metabolism. From the viewpoint of developmental biology, several recent studies have clearly indicated that mitochondria are functionally and morphologically reorganized for adaptation to an embryonic stem cell (ESC)-like intracellular environment during induced pluripotent stem cell (iPSC) generation.^1, 2, 3, 4, 5, 6^ Moreover, mtDNA haplogroups (i.e., genetic population groups that share a common ancestor), which are known to be associated with various phenotypes (e.g., disease susceptibility, environmental adaptation or aging), also affect their intrinsic gene expression signatures involved in pluripotency, differentiation, DNA methylation and mitochondrial energy metabolism.^7^ Thus, appropriate mitochondrial rejuvenation or maturation may be one important step for *bona fide* cellular reprogramming or differentiation, as well as for epigenetic modification or resetting in nuclear DNA.

Most parts of pathogenic mutations in mtDNA-specific tRNA genes responsible for various types of mitochondrial diseases have been reported as heteroplasmy (i.e., wild-type mtDNA and mutant mtDNA coexist within a single cell), and induced mitochondrial dysfunction emerges only when mutation ratios of mtDNA exceed their intrinsic pathogenic thresholds at a cellular level.^8^ Mitochondrial diseases caused by heteroplasmic mtDNA mutations present a wide variety of affected tissues and organs (e.g., central nervous system or cardiovascular system) among patients,^9, 10^ probably due to variations in mutant mtDNA proportions at each tissue and organ level. Therefore, disease-relevant iPSCs carrying heteroplasmic mtDNA mutations will greatly help us to open new avenues for studying the patient-specific definitive genotype–phenotype relationship of affected tissues and organs in mitochondrial diseases.^11^ In fact, several groups and we have reported the generation and the application of patient-derived iPSCs carrying various heteroplasmic mtDNA mutations toward *in vitro* human mitochondrial disease modeling;^12, 13, 14, 15, 16, 17, 18^ however, it remains uncertain whether and how such heteroplasmic mtDNA mutations affect particular cellular fate-determination processes during development. Recently, we also demonstrated that mitochondrial respiratory dysfunction caused by a heteroplasmic m.3243 A>G mutation in *MT-TL1* gene,^19^ which is the most representative mutant mtDNA, strongly inhibits cellular reprogramming but does not affect maintenance of the pluripotent state.^20^ Our findings may indicate that the degree of the molecular pathogenic influence of heteroplasmic mtDNA mutations actually changes during cellular lineage-commitment processes along with the degree of functional maturation in mitochondria.

In this study, we established two isogenic iPSC lines carrying different proportions of m.3243 A>G from the same patient; one exhibited apparently normal and the other showed most likely impaired mitochondrial respiratory function. Using these isogenic iPSC lines, we demonstrated that induced mitochondrial respiratory dysfunction triggered by high proportions of m.3243 A>G strongly inhibits maturation and survival of iPSC-derived neurons and cardiomyocytes. Such *in vitro* neuronal and cardiac maturation defects were also confirmed by using another patient-derived iPSC line carrying quite high proportion of m.3243 A>G. Our presenting data therefore demonstrate that isogenic iPSC lines with different proportions of m.3243 A>G would make enormous contributions as *in vitro* human cellular disease models to greatly facilitate iPSC-based drug discovery and regenerative therapeutics in mitochondrial diseases.

## Results

### Generation of patient-derived isogenic iPSC lines carrying different proportions of m.3243A>G

**Figure 1:**
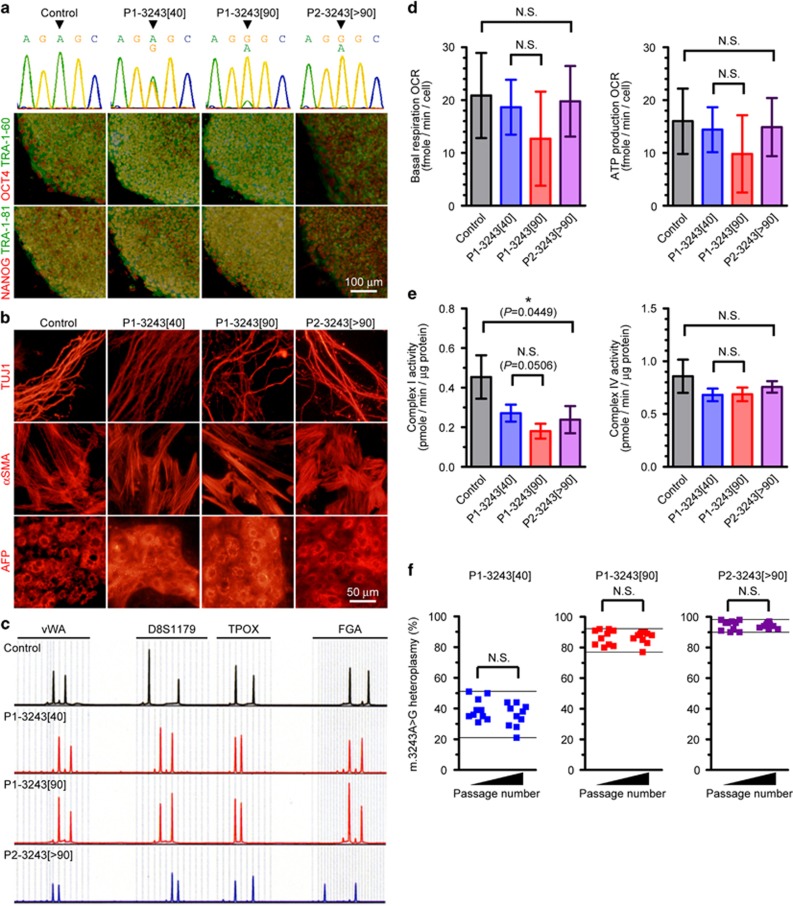
Generation of patient-derived isogenic iPSC lines carrying different proportions of m.3243A>G. (**a**) Representative images of the established iPSC lines; OCT4 (red), NANOG (red), TRA-1-60 (green) and TRA-1-81 (green). Electropherogram of heteroplasmic m.3243A>G mutation in each iPSC line was also shown. Arrowheads indicate m.3243A>G. (**b**) Representative images of the embryoid body (EB)-mediated *in vitro* spontaneous differentiation; TUJ1 (ectoderm, red), *α*SMA (mesoderm, red) and AFP (endoderm, red). (**c**) Representative images of STR variations (4 out of 16 genetic loci analyzed) demonstrated that isogenic iPSC lines carrying different proportions of m.3243A>G (P1-3243[40] and P1-3243[90]) shared the same nuclear DNA genetic background. (**d**) Mitochondrial respiratory function of patient-derived iPSC lines. Oxygen consumption rate (OCR) of each iPSC line was measured by a flux analyzer. Biological replicates of each iPSC line used were as follows: Control (*n*=5), P1-3243[40] (*n*=3), P1-3243[90] (*n*=5), P2-3243[>90] (*n*=4). Statistical significance was evaluated by unpaired, two-tailed *t*-test. NS, not significant. (**e**) Mitochondrial respiratory chain complexes activity of patient-derived iPSC lines. Three biological replicates of each iPSC line were used for the measurements. Statistical significance was evaluated by unpaired, two-tailed *t*-test. *P<0.05, NS, not significant. (**f**) Time-dependent changes in the distributions of m.3243A>G proportions in patient-derived iPSC line at each single-iPSC-colony level. Statistical significance was evaluated by unpaired, two-tailed *t*-test. NS, not significant

First, we generated two isogenic iPSC lines from the same patient, each of which possessed different proportions of m.3243A>G (approximately 40 and 90% proportions of mutant mtDNA; denoted as P1-3243[40] and P1-3243[90], respectively). We also established two additional iPSC lines, each of which were derived from healthy control subject (denoted as Control) and from another patient carrying over 90% proportion of m.3243A>G (denoted as P2-3243[>90]), respectively. We have previously reported that the molecular pathogenic threshold level of m.3243A>G with regard to mitochondrial respiratory function is ~90% in patient-derived clonal fibroblasts.^20^ We confirmed that no marked difference was observed between all iPSC lines with regard to ESC-like pluripotent characteristics such as pluripotency markers expression and embryoid body (EB)-mediated *in vitro* spontaneous differentiation into three germ layers (Figures 1a and b), in addition to pluripotency genes expression and silenced transgenes expression (Supplementary Figures S1A and B). Genetic identity of these isogenic iPSC lines was also verified by analysis of short tandem repeat variations (Figure 1c). We measured the overall mitochondrial respiration profile of all iPSC lines by a flux analyzer. Although no statistical significance was observed between two isogenic iPSC lines (P1-3243[40] and P1-3243[90]), mitochondrial energy metabolic potentials (e.g., basal respiration and ATP production) of P1-3243[90] iPSC line were both lower than those of P1-3243[40] iPSC line (Figure 1d). We further analyzed enzymatic activities of mitochondrial respiratory chain complexes in all iPSC lines. In fact, mitochondrial respiratory chain complex I activity was significantly suppressed by over 90% proportion of m.3243A>G (P2-3243[>90] vs Control), whereas mitochondrial respiratory chain complex IV activity was apparently unaffected in all iPSC lines (Figure 1e). Although no statistical significance was observed between two isogenic iPSC lines (P1-3243[40] and P1-3243[90]), mitochondrial respiratory chain complex I activity of P1-3243[90] iPSC line was actually lower than that of P1-3243[40] iPSC line. We also randomly selected several iPSC colonies from each patient-derived iPSC line to determine m.3243A>G proportions at each single-iPSC-colony level and found no significant segregation in m.3243A>G proportions during self-renewal of iPSCs throughout this study (i.e., at least 5–10 passages in culture of each iPSC line) (Figure 1f). We therefore concluded that two isogenic iPSC lines with different proportions of m.3243 A>G from the same patient (P1-3243[40] and P1-3243[90]) were successfully established; one exhibited apparently normal and the other showed most likely impaired mitochondrial respiratory function.

### Inhibited cardiac maturation triggered by exceeding the pathogenic threshold level of m.3243A>G

**Figure 2:**
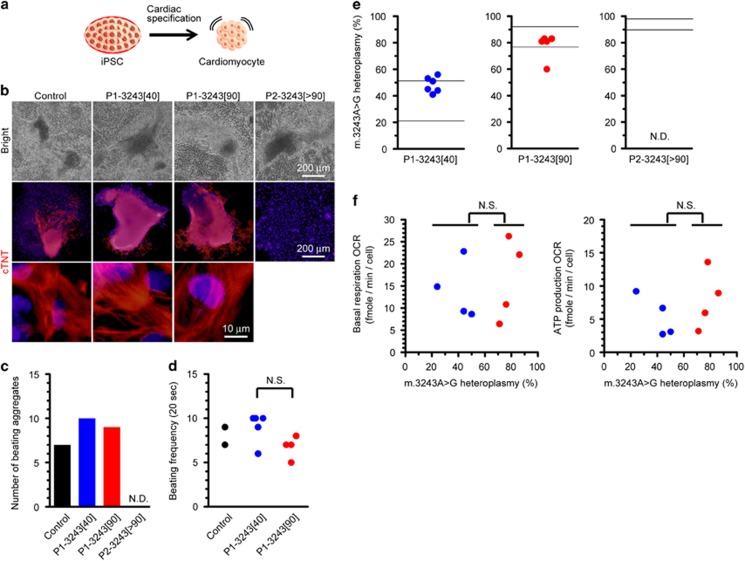
Induced cardiac maturation defects triggered by exceeding the pathogenic threshold level of m.3243A>G. (**a**) Experimental design used to identify the molecular pathogenic influence of m.3243A>G on cardiac differentiation. (**b**) Representative images of cardiomyocytes derived from each patient-derived iPSC line; cTNT (red). Cell nuclei were co-stained with Hoechst 33342 (blue). No cTNT-positive cardiomyocytes were observed in P2-3243[>90] iPSC line. (**c**) Total number of beating aggregates after cardiac differentiation in each patient-derived iPSC line. Cardiac induction was independently performed three times, and data were gathered for graph preparation. ND, not detected. (**d**) Beating frequency of cardiomyocytes derived from each patient-derived iPSC line. Statistical significance was evaluated by unpaired, two-tailed *t*-test. NS, not significant. Representative movies of beating cardiomyocytes derived from each patient-derived iPSC line were also shown in Supplementary Movies S1–S3, respectively. (**e**) The distributions of m.3243A>G proportions in cardiomyocytes derived from patient-derived iPSC lines. Immunostained cells were collected for further mtDNA mutation analysis. ND, not detected. (**f**) Relationship between the distributions of m.3243A>G proportions and mitochondrial respiratory function in cardiomyocytes derived from patient-derived isogenic iPSC lines. Oxygen consumption rate (OCR) of beating cardiomyocytes was measured by a flux analyzer. The proportions of m.3243A>G in cardiomyocytes were determined after biochemical measurement. Biological replicates of beating cardiomyocytes used were as follows: P1-3243[40] (*n*=4), P1-3243[90] (*n*=4). Statistical significance was evaluated by unpaired, two-tailed *t*-test. NS, not significant

Next, we asked whether and how heteroplasmy levels of m.3243A>G affect cardiac maturation (Figure 2a). Using Control iPSC line, we confirmed the successful specification into cTNT-positive beating cardiomyocytes. P1-3243[90] iPSC line, which exhibited most likely impaired mitochondrial respiratory function, was also able to differentiate into beating cardiomyocytes expressing the representative cardiac lineage marker of cTNT similarly to those of isogenic P1-3243[40] iPSC line; in contrast, no beating cardiomyocytes were obtained from P2-3243[>90] iPSC line, which exhibited impaired mitochondrial respiratory function (Figures 2b–d and Supplementary Movies S1–S3). Of note, no marked difference was observed during the time course of cardiac induction between these iPSC lines (see also Figure 2b). Interestingly, however, all cardiomyocytes derived from P1-3243[90] iPSC line possessed less than 90% proportions of m.3243 A>G (Figure 2e). To confirm the molecular pathogenic influence of m.3243A>G on cardiac lineage commitment, we measured mitochondrial respiratory function of iPSC-derived cardiomyocytes by a flux analyzer; as expected, cardiomyocytes derived from P1-3243[90] iPSC line, all of which exhibited below the molecular pathogenic threshold level of m.3243A>G, showed apparently normal mitochondrial respiration profile and mitochondrial energy metabolic potentials (e.g., basal respiration and ATP production) similarly to those derived from P1-3243[40] iPSC line (Figure 2f). In addition, we found that one out of five iPSC-derived cardiomyocytes showed a significant decrease in m.3243A>G heteroplasmy level (approximately 60% proportion of mutant mtDNA) when compared with the distributions of m.3243A>G heteroplasmy levels in the parental P1-3243[90] iPSC line (77–92% proportions of mutant mtDNA) (see also Figure 2e). We also added mtDNA copy number analysis for iPSC-derived cardiomyocytes and their parental iPSCs. Cardiomyocytes derived from two isogenic iPSC lines (P1-3243[40] and P1-3243[90]) had more mtDNA copies per cell than those in the parental iPSCs; however, no significant difference in mtDNA copy number was observed between two isogenic iPSC lines (P1-3243[40] and P1-3243[90]), or among their iPSC-derived cardiomyocytes (Supplementary Figure S2). We therefore concluded that mitochondrial respiratory dysfunction caused by exceeding the pathogenic threshold level of m.3243A>G induced cardiac maturation defects.

### Induced neuronal cell death triggered by exceeding the pathogenic threshold level of m.3243A>G

**Figure 3:**
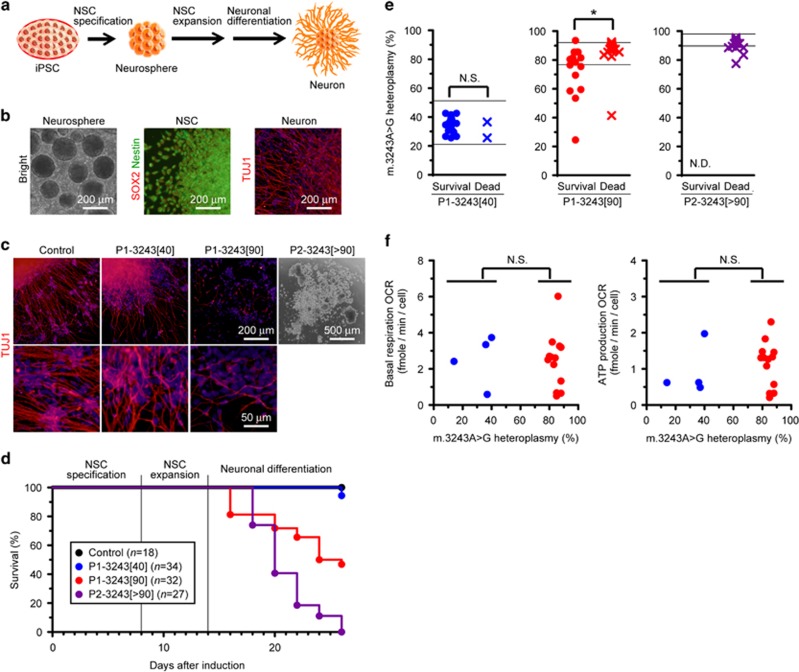
Induced neuronal cell death triggered by exceeding the pathogenic threshold level of m.3243A>G. (**a**) Experimental design used to identify the molecular pathogenic influence of m.3243A>G on neuronal differentiation. (**b**) Representative images of successive differentiation into neurons via neural stem cell (NSC) specification and expansion using Control iPSC line; SOX2 (red), Nestin (green), TUJ1 (red). Cell nuclei were co-stained with Hoechst 33342 (blue). (**c**) Representative images of neurons derived from each patient-derived iPSC line; TUJ1 (red). Cell nuclei were co-stained with Hoechst 33342 (blue). Representative image of induced neuronal cell death during neuronal differentiation in P2-3243[>90] iPSC line was also shown. (**d**) Induced neuronal cell death, but stable NSC specification and expansion, in two patient-derived iPSC lines carrying high m.3243A>G proportions (P1-3243[90] and P2-3243[>90]). Neuronal differentiation was independently performed three times, and data were gathered for graph preparation. (**e**) The distributions of m.3243A>G proportions in neurons derived from patient-derived iPSC lines. Immunostained 'survival' cells and spontaneously detached 'dead' neurospheres were collected for further mtDNA mutation analysis. Notably, 2 out of 34 neurospheres derived from P1-3243[40] iPSC line were detached from culture surfaces just before immunostaining (see also **d**). Therefore, we evaluated these detached neurospheres as 'dead' in this experiment. Statistical significance was evaluated by unpaired, two-tailed *t*-test. *P<0.05, NS, not significant; ND, not detected. (**f**) Relationship between the distributions of m.3243A>G proportions and mitochondrial respiratory function in neurons derived from patient-derived isogenic iPSC lines. Oxygen consumption rate (OCR) of neurons was measured by a flux analyzer. The proportions of m.3243A>G in neurons were determined after biochemical measurement. Biological replicates of neurons used were as follows: P1-3243[40] (*n*=4), P1-3243[90] (*n*=13). Statistical significance was evaluated by unpaired, two-tailed *t*-test. NS, not significant

We then differentiated these iPSC lines into neurons using our stepwise induction method to clarify whether and how heteroplasmy levels of m.3243 A>G also affect neuronal maturation (Figure 3a). Using Control iPSC line, we confirmed that our neuronal induction protocol showed highly efficient neural stem cell (NSC) specification and neuronal differentiation (Figure 3b). Although P1-3243[40] iPSC line showed no apparent influence of m.3243 A>G on directed differentiation into TUJ1-positive neurons similarly to that of Control iPSC line, two other iPSC lines carrying high proportions of m.3243 A>G (P1-3243[90] and P2-3243[>90]) showed induced cell death during neuronal lineage commitment; in particular, poorly surviving neurons in P1-3243[90] iPSC line, which exhibited most likely impaired mitochondrial respiratory function, and no living neurons in P2-3243[>90] iPSC line, which exhibited impaired mitochondrial respiratory function, were observed, respectively (Figures 3c and d). We also evaluated the completely detached and collapsed neurospheres in P2-3243[>90] iPSC line as 'dead' in this experiment (see also Figure 3c). Of note, no cell death was observed during NSC specification and expansion in these iPSC lines, suggesting that m.3243 A>G has minimal molecular pathogenic influence on NSCs. Focusing on P1-3243[90] iPSC line, m.3243 A>G heteroplasmy levels were significantly higher in 'dead' neurospheres (86±12% proportions of mutant mtDNA) than those in 'survival' neurons (73±17% proportions of mutant mtDNA) with statistical significance (Figure 3e). To confirm the molecular pathogenic influence of m.3243 A>G on neuronal lineage commitment, we measured mitochondrial respiratory function of iPSC-derived 'survival' neurons by a flux analyzer; similarly to the case of iPSC-derived cardiomyocytes, 'survival' neurons derived from P1-3243[90] iPSC line, all of which possessed less than 90% proportions of m.3243 A>G, showed apparently normal mitochondrial respiration profile and mitochondrial energy metabolic potentials (e.g., basal respiration and ATP production) similarly to those derived from P1-3243[40] iPSC line (Figure 3f). More remarkable than the case of cardiac lineage commitment, some iPSC-derived 'survival' neurons also showed drastic decreases in m.3243 A>G heteroplasmy levels (i.e., 5 out of 15 neurons exhibited less than 70% proportions of mutant mtDNA) when compared with the distributions of m.3243 A>G heteroplasmy levels in the parental P1-3243[90] iPSC line (77–92% proportions of mutant mtDNA) (see also Figure 3e), suggesting that mutant mtDNA segregation may occur in some cell populations during neuronal maturation process in a stochastic manner. We further prepared other lines of iPSC-derived neurons from the parental P1-3243[90] iPSC line to experimentally reproduce such mutant mtDNA segregation behavior and to clarify the relationship between the segregation of m.3243 A>G heteroplasmy levels and the changes in mtDNA copy number. Unfortunately, however, no significant segregation of m.3243 A>G heteroplasmy levels was observed during the repetitive neuronal differentiation assays. In contrast to the case of iPSC-derived cardiomyocytes, 'survival' neurons derived from two isogenic iPSC lines (P1-3243[40] and P1-3243[90]) possessed less mtDNA copies per cell than those in the parental iPSCs; however, no significant difference in mtDNA copy number was observed between two isogenic iPSC lines (P1-3243[40] and P1-3243[90]), or among their iPSC-derived neurons (Supplementary Figure S3). We therefore concluded that mitochondrial respiratory dysfunction caused by exceeding the pathogenic threshold level of m.3243A>G also induced neuronal cell death; this phenomenon is similar to, but more pronounced than, that in cardiac lineage.

### Neuronal maturation defect was also recapitulated by using mtDNA-depleted neuroblastoma cells

**Figure 4:**
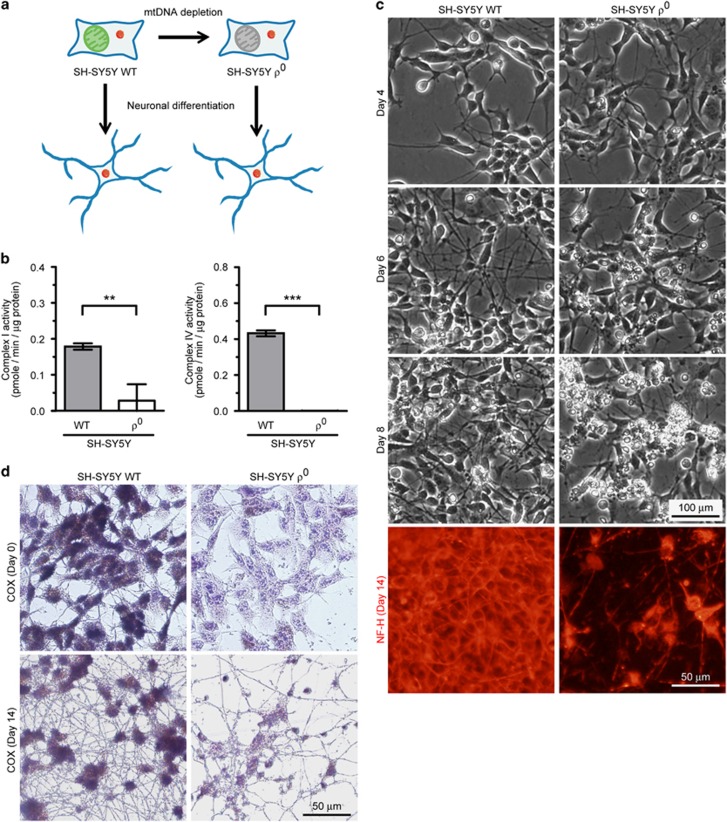
mtDNA-depleted neuroblastoma cells recapitulate neuronal maturation defect and even neuronal cell death during neuronal lineage commitment. (**a**) Experimental design used to identify whether mtDNA-depleted neuroblastoma cells recapitulate neuronal maturation defect and even neuronal cell death during neuronal lineage commitment. (**b**) Mitochondrial respiratory chain complexes activity of neuroblastoma cell lines. Three biological replicates of SH-SY5Y WT and SH-SY5Y *ρ*^0^ were used for the measurements. Statistical significance was evaluated by unpaired, two-tailed *t*-test. **P<0.01, ***P<0.001. (**c**) Representative images of neurons derived from SH-SY5Y WT and SH-SY5Y *ρ*^0^; NF-H (red). Marked neuronal cell death in SH-SY5Y *ρ*^0^ was also observed at day 8. Representative movies of differentiating neurons derived from SH-SY5Y WT and SH-SY5Y *ρ*^0^ were also shown in Supplementary Movies S4 and S5, respectively. (**d**) Representative images of cytochemical COX staining for undifferentiated and differentiated SH-SY5Y WT and SH-SY5Y *ρ*^0^; COX (brown). Cell nuclei were co-stained with hematoxylin (purple). Both samples of SH-SY5Y WT and SH-SY5Y *ρ*^0^ were stained simultaneously for the same period

We further addressed whether severe mitochondrial respiratory dysfunction, which is triggered by mtDNA depletion, is also able to recapitulate neuronal maturation defect and even neuronal cell death during neuronal lineage commitment. We used SH-SY5Y neuroblastoma cell line (SH-SY5Y WT) to prepare its mtDNA-depleted cell line (SH-SY5Y *ρ*^0^) and to differentiate both neuroblastoma cell lines into neurons (Figure 4a). We confirmed, in advance, that SH-SY5Y *ρ*^0^ line showed severe mitochondrial respiratory dysfunction triggered by mtDNA depletion (Figure 4b). In fact, cytochemical staining of cytochrome *c* oxidase (COX), an indicator of mitochondrial respiration activity, also indicated that SH-SY5Y WT line showed strongly COX-positive, whereas SH-SY5Y *ρ*^0^ line showed COX-negative (Figure 4d). As we expected, neuronal maturation was markedly suppressed by severe mitochondrial respiratory dysfunction triggered by mtDNA depletion, and in some cells, induced neuronal cell death was also observed in SH-SY5Y *ρ*^0^ line during neuronal lineage commitment (Figure 4c and Supplementary Movies S4 and S5) with similar trend to iPSC-derived neurons carrying high proportions of m.3243 A>G. In this case, the remaining 'survival' neurons in SH-SY5Y *ρ*^0^ line were COX-negative (Figure 4d). Although neuroblastoma cells have several distinct genetic, epigenetic and energy metabolic properties from iPSCs, we concluded that mitochondrial respiratory dysfunction caused by defective mtDNA with various mutation types actually induced neuronal maturation defect and even neuronal cell death *in vitro*.

## Discussion

**Figure 5:**
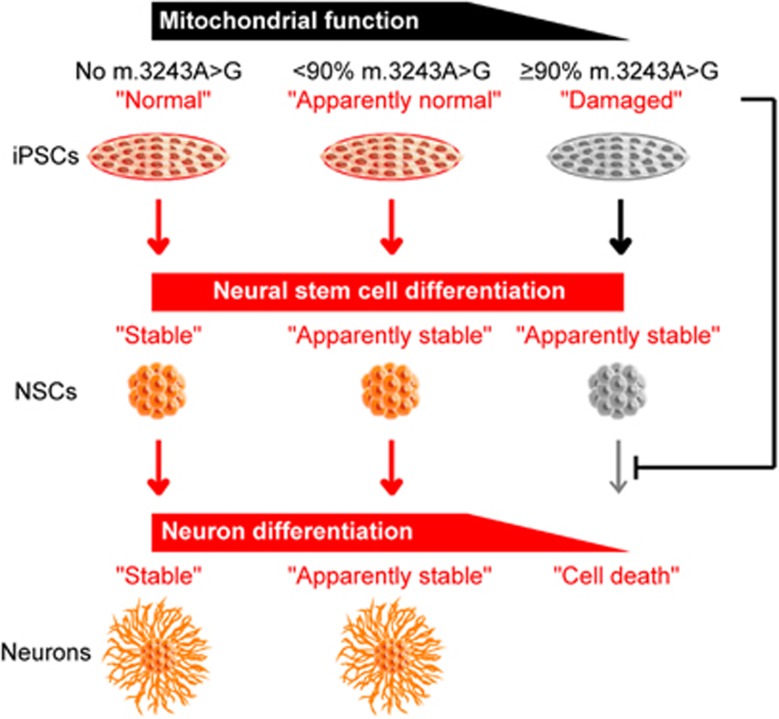
Graphical summary showing the relationship between mtDNA pathogenic threshold and inhibited neuronal differentiation. iPSC lines carrying over the pathogenic threshold level of m.3243A>G showed neuronal maturation defect and even neuronal cell death during neuronal lineage commitment; however, no cell death was observed during NSC specification and expansion in these iPSC lines, suggesting that m.3243A>G has minimal molecular pathogenic influence on NSCs

In this study, we generated two isogenic iPSC lines from the same patient; one exhibited apparently normal and the other showed most likely impaired mitochondrial respiratory function. Using these isogenic iPSC lines, our lineage-specific directed differentiation methods demonstrated that induced mitochondrial respiratory dysfunction triggered by high proportions of m.3243 A>G strongly inhibits maturation and survival of iPSC-derived neurons and cardiomyocytes. Such *in vitro* maturation defects in both neuronal and cardiac lineages were also confirmed using another patient-derived iPSC line carrying over 90% proportion of m.3243 A>G. In addition to our results, Hämäläinen *et al.*^15^ reported the pathogenic influences of a heteroplasmic m.3243 A>G mutation on neuronal differentiation; briefly, their established patient-origin iPSC-derived neurons carrying approximately 80–85% proportions of m.3243 A>G exhibited specific downregulation of mitochondrial respiratory chain complex I at both transcript and protein levels and showed accelerated mitophagy via the PARKIN–PINK1 pathway, probably due to clearance of damaged mitochondria for further neuronal differentiation and maturation. Taking these previous findings with our presenting data, we propose that appropriate mitochondrial rejuvenation or maturation must be required for *bona fide* cellular reprogramming or differentiation, and the degree of molecular pathogenic influence of mutant mtDNA actually determines the severity of the cell-type-specific disease phenotypes *in vitro*, including the differentiation efficiency into particular cell types (Figure 5).

As we noted above, severe mitochondrial respiratory dysfunction strongly induces neuronal cell death *in vitro*; however, most parts of mitochondrial disease patients carrying mutant mtDNA undergo normal brain development *in vivo* before symptomatic appearance. What is the crucial difference between iPSC-based *in vitro* cellular disease phenotypes and *in vivo* clinical symptoms? Some previous molecular neuropathological studies using postmortem brain of mitochondrial disease patients found that neuronal cells carrying higher proportions of mutant mtDNA frequently remained in some patients' cerebellar lesions (e.g., dentate nucleus neurons, olivary neurons and Purkinje cells).^21, 22^ These findings suggest that neuronal cell death does not always correlate with mutant mtDNA proportions, leading to the discrepancy between our iPSC-based *in vitro* recapitulation of neuronal development and *in vivo* brain pathology of mitochondrial disease patients. With regard to this discrepancy, it is hypothesized that physiological and physical interaction with other non-neuronal cell types in the brain (e.g., astrocytes) may strongly enhance maturation and long-term survival of neurons having damaged mitochondria. Astrocytes are known to play a role as an energy supplier to neurons through the release of lactate;^23^ for example, a co-culture system with astrocytes is generally used for accelerated functional maturation and long-term survival of neurons *in vitro*. Moreover, the predominant energy metabolic system in astrocytes is glycolysis, while that in neurons is mitochondrial respiration,^23^ suggesting no apparent influence of mitochondrial respiration defects on physiological function in astrocytes to support neurons. In fact, we displayed the successive observation of TUJ1-positive neurons derived from P2-3243[>90] iPSC line through EB-mediated *in vitro* spontaneous differentiation, and even this iPSC line did not produce neurons using the directed neuronal differentiation method. On the other hand, aberrant early embryogenesis was reported using fertilized eggs derived from a female mito-mouse carrying 70% proportion of 4696- bp mtDNA deletion;^24^ this mouse mutant mtDNA exhibited the intrinsic pathogenic threshold level (60–80% proportions of mutant mtDNA) in relation to mitochondrial respiratory function, which was confirmed by *in vitro* biochemical analysis using trans-mitochondrial cellular systems and by *in vivo* phenotypic analysis using several lines of mito-mice.^25^ Although there are some experimental differences between their findings and our presenting data (iPSC-based human model vs mouse model, mtDNA point mutation vs mtDNA partial deletion, etc.), the defective *in vitro* differentiation into particular cell types triggered by severely impaired mitochondrial respiration may be suggestive of such *in vivo* embryonic lethality.

Mitochondrial diseases present a broad clinical spectrum even among patients carrying the same heteroplasmic mtDNA mutations (e.g., variations in age of onset, in affected tissues and organs, or in disease progression and phenotypic severity), and vice versa, different mtDNA mutations share similar clinical features in mitochondrial diseases. Such clinical phenotypic diversity frequently makes us complicated to understand the overall pathology of mitochondrial diseases; therefore, curable treatments have yet to be established. Thus, our established isogenic iPSC lines from the same mitochondrial disease patient exhibiting either apparently normal or impaired mitochondrial respiratory function must be promising tools not only to recapitulate tissue- and organ-specific disease phenotypes but also to efficiently explore candidate chemical compounds (i) that ameliorate mitochondrial respiratory dysfunction or (ii) that induce reduced mutant mtDNA proportions. We believe that our presenting data display new insights not only into understanding how mitochondrial respiratory dysfunction triggered by heteroplasmic mtDNA mutations influences cellular fate-determining processes but also into facilitating the applications in future iPSC-based drug discovery and regenerative therapeutics in mitochondrial diseases.

## Materials and Methods

### Patients

This study was approved by NCNP Institutional Review Board and was stringently conducted in accordance with the ethical principles of the 'Declaration of Helsinki'. Patient biopsy was performed for diagnostic purposes only after we received written informed consent with permission to study patient-derived iPSCs.

### Fibroblast culture

Primary fibroblasts were established from patient-derived skin biopsies via a standard protocol. Patient-derived fibroblasts were maintained in DMEM/F12 (Gibco, Waltham, MA, USA) supplemented with 10% FBS (Gibco), 100 units/ml penicillin (Gibco), 100 *μ*g/ml streptomycin (Gibco) at 37 °C under humidified atmosphere of 5% CO_2_. Culture medium was changed every 3 days. During establishment of primary fibroblasts, 0.5 *μ*g/ml MC210 (DS Pharm, Osaka, Japan) as a mycoplasmacidal reagent and 2.5 *μ*g/ml fungizone (Gibco) as a fungicidal reagent were also added to culture medium.

### Generation of patient-derived iPSCs with episomal vector

Patient-derived iPSCs were generated using episomal vectors as described elsewhere^26^ with modifications: briefly, each 1 *μ*g of episomal plasmid vectors (Plasmid #27077, #27078, #27080; Addgene, Cambridge, MA, USA) were electroporated into patient-derived myoblasts (5 × 10^5^ cells) with an electroporator (Neon; Invitrogen, Waltham, MA, USA). Transformed patient-derived myoblasts (1 × 10^5^ cells) were reseeded onto mouse embryonic fibroblasts (MEF; ReproCELL, Yokohama, Japan) 4 days after electroporation. The next day, culture medium was replaced with primate ESC culture medium (ReproCELL) supplemented with 10 ng/ml bFGF (ReproCELL), 100 units/ml penicillin (Gibco), 100 *μ*g/ml streptomycin (Gibco) and transformed patient-derived myoblasts were maintained at 37 °C under humidified atmosphere of 5% CO_2_. Culture medium was changed every other day. Emergent colonies with ESC-like morphology were manually picked up to establish patient-derived iPSCs, and these iPSCs were expanded either on MEF-seeded dishes in primate ESC culture medium or on Geltrex (Gibco)-coated dishes in mTeSR1 medium (StemCell Technologies, Vancouver, BC, Canada) supplemented with 100 units/ml penicillin (Gibco) and 100 *μ*g/ml streptomycin (Gibco) for long-term maintenance. Culture medium was changed daily.

To evaluate the distributions of m.3243 A>G proportions in each patient-derived iPSC line, we randomly picked up several iPSC colonies from each patient-derived iPSC line to extract DNA for determination of m.3243 A>G proportions at each single-iPSC-colony level.

### Characterization of patient-derived iPSCs

Characterization of patient-derived iPSCs via detection of pluripotency markers was performed according to our previous report:^17^ Briefly, cultured and harvested patient-derived iPSCs were transferred onto MEF-seeded multi-well culture plates and were maintained in primate ESC culture medium at 37 °C under humidified atmosphere of 5% CO_2_. Culture medium was changed daily. After 3 days in culture, patient-derived iPSCs were characterized by standard immunocytochemical protocol. Fluorophore-conjugated primary antibodies used were as follows: Cy3-conjugated anti-OCT4 (1:100 dilution; Millipore, Billerica, MA, USA), Cy3-conjugated anti-NANOG (1:100 dilution; Millipore), AlexaFluor 488-conjugated anti-TRA-1-60 (1:100 dilution; Millipore), AlexaFluor 488-conjugated anti-TRA-1-81 (1:100 dilution; Millipore). Stained samples were observed under a fluorescent microscope (IX71 System; Olympus, Tokyo, Japan).

*in vitro* spontaneous differentiation of patient-derived iPSCs into EB-mediated three germ layers was also performed according to our previous report:^17^ Briefly, cultured and harvested patient-derived iPSCs were transferred onto ultra-low-adherent culture dishes (HydroCell; CellSeed, Tokyo, Japan) and were maintained in primate ESC culture medium without bFGF at 37 °C under humidified atmosphere of 5% CO_2_. Culture medium was changed every other day. After 7 days in floating culture, emergent EBs were transferred onto Geltrex (Gibco)-coated multi-well culture plates and were maintained in primate ESC culture medium without bFGF at 37 °C under humidified atmosphere of 5% CO_2_. Culture medium was changed every other day. After 14 additional days in adherent culture, spontaneously differentiated cells were characterized by standard immunocytochemical protocol. Primary antibodies used were as follows: anti-TUJ1 (1:200 dilution; Abcam, Cambridge, UK), anti-*α*SMA (1:40 dilution; Abcam), anti-AFP (1:200 dilution; Abcam). Secondary antibody used was AlexaFluor 568 (1:800 dilution; Molecular Probes, Waltham, MA, USA). Stained samples were observed under a fluorescent microscope (IX71 System; Olympus).

Short tandem repeat (STR) analysis was performed to confirm the genetic identity of the established isogenic iPSC lines: Briefly, extracted DNA as template (0.5 ng) was amplified using a thermal cycler (GeneAmp PCR System 9700; Applied Biosystems, Waltham, MA, USA) with a PowerPlex 16 HS System kit (Promega, Fitchburg, WI, USA) according to the manufacturer's instructions. The amplified DNA fragments were electrophoresed using a DNA analyzer (ABI PRISM 3130xl; Applied Biosystems). The obtained data were analyzed using GeneMapper Software (Ver. 5.0; Applied Biosystems).

### Analysis of mtDNA mutation

Long PCR-based whole-mtDNA sequencing for the patient was performed as described elsewhere^27^ with modifications to eliminate any adverse results arising from pseudo-sequences in nuclear DNA: Briefly, extracted DNA as a template (10 ng) was amplified via mtDNA-specific long-range PCR and the following mtDNA-specific nested PCR using a thermal cycler (GeneAmp PCR System 9700; Applied Biosystems). The amplified mtDNA fragments were sequenced using a DNA analyzer (ABI PRISM 3130xl; Applied Biosystems).

Pyrosequencing was performed to determine m.3243 A>G proportions: Briefly, extracted DNA as a template (10–20 ng) was amplified using a thermal cycler (GeneAmp PCR System 9700; Applied Biosystems). The amplified mtDNA fragments were sequenced using a pyrosequencing instrument (PyroMark Q24 Advanced; Qiagen, Venlo, Netherlands) with a PyroMark Q24 Advanced Reagents kit (Qiagen) according to the manufacturer's instructions. The obtained data were analyzed using PyroMark Q24 Advanced Software (Ver. 3.0.0; Qiagen). Primers used are listed in Supplementary Table S1.

### Analysis of mtDNA copy number

mtDNA copy number analysis was performed according to our previous report:^20^ Briefly, extracted DNA as a template (1 ng) was used for quantitative PCR with a SYBR Green I PCR Master Mix kit (Roche, Basel, Switzerland) according to the manufacturer's instructions. A real-time PCR system (LightCycler 480II; Roche) was used to measure mtDNA copy number per cell. Measurement for each sample was performed in triplicate. ΔΔC_T_-based relative quantification method was adopted for data analysis. Primers used are listed in Supplementary Table S1.

### Analyses of pluripotency genes expression and transgenes silencing

Reverse transcription was performed with PrimeScript RT Master Mix kit (TaKaRa Bio, Shiga, Japan) according to the manufacturer's instructions. After reverse transcription of extracted total RNA, total cDNA as a template (10 ng) was used for quantitative PCR with a SYBR Green I PCR Master Mix kit (Roche) according to the manufacturer's instructions. A real-time PCR system (LightCycler 480II; Roche) was used to measure pluripotency genes expression and transgenes silencing. Measurement for each sample was performed in triplicate. ΔΔC_T_-based relative quantification method was adopted for data analysis. Primers used are listed in Supplementary Table S1.

### Directed differentiation of iPSCs into cardiomyocytes

Directed differentiation of patient-derived iPSCs into cardiomyocytes was performed as described elsewhere^28^ with modifications: Briefly, patient-derived iPSCs were cut into uniform-sized pieces of colonies using the STEMPRO EZ Passage (Invitrogen) to transfer onto Geltrex (Gibco)-coated culture dishes and were maintained in mTeSR1 medium (StemCell Technologies) at 37 °C under humidified atmosphere of 5% CO_2_. Culture medium was changed daily. After 7 days in culture, culture medium was switched to Cardiac induction medium I (RPMI 1640 medium (Gibco) supplemented with 1 × B27 minus insulin (Gibco), 100 units/ml penicillin (Gibco), 100 *μ*g/ml streptomycin (Gibco), 100 ng/ml Activin A (Peprotech, Rocky Hill, NJ, USA)) for first 1 day, Cardiac induction medium II (RPMI 1640 medium (Gibco) supplemented with 1 × B27 minus insulin (Gibco), 100 units/ml penicillin (Gibco), 100 *μ*g/ml streptomycin (Gibco), 10 ng/ml BMP4 (Peprotech), 10 ng/ml bFGF (Peprotech)) for next 4 days, and Cardiac induction medium III (RPMI 1640 medium (Gibco) supplemented with 1 × B27 minus insulin (Gibco), 100 units/ml penicillin (Gibco), 100 *μ*g/ml streptomycin (Gibco), 100 ng/ml DKK-1 (Peprotech)) for further 6 days, sequentially. Culture medium was changed every other day. Culture medium was finally switched to Cardiac maturation medium (RPMI 1640 medium (Gibco) supplemented with 1 × B27 minus insulin (Gibco), 100 units/ml penicillin (Gibco), 100 *μ*g/ml streptomycin (Gibco)) and was changed every other day for terminal differentiation. The beating aggregates began to emerge at around 15 days of cardiac differentiation. At 26 or 27 days of cardiac differentiation in total, each beating cardiomyocyte-aggregate was transferred onto each well of Geltrex (Gibco)-coated multi-well culture plates and were used for further analyses.

Emergent cardiomyocytes were maintained in cardiac maturation medium and were characterized according to standard immunocytochemical protocol. Primary antibody used was as follows: anti-cTNT (1:200 dilution; ThermoFisher Scientific, Waltham, MA, USA). Secondary antibody used was as follows: AlexaFluor 568 (1:800 dilution; Molecular Probes). Stained samples were observed under a fluorescent microscope (IX71 System; Olympus).

### Directed differentiation of iPSCs into neurons

Directed differentiation of patient-derived iPSCs into neurons was performed as follows: Briefly, cultured and harvested patient-derived iPSCs were transferred onto ultra-low-adherent culture dishes (HydroCell; CellSeed) and were maintained in NSC specification medium (Essential 6 medium (Gibco) supplemented with 100 units/ml penicillin (Gibco), 100 *μ*g/ml streptomycin (Gibco), 10 *μ*M SB431542 (Wako, Osaka, Japan), 100 nM LDN193189 (Wako)) for first 8 days at 37 °C under humidified atmosphere of 5% CO_2_. Culture medium was changed every other day. After floating culture, each neurosphere was transferred onto each well of Geltrex (Gibco)-coated multi-well culture plates and were maintained in NSC expansion medium (1:1 mixture of DMEM/F12 (Gibco) and Neurobasal medium (Gibco) supplemented with 1 × N2 (Gibco), 1 × B27 minus vitamin A (Gibco), 1 × GlutaMAX (Gibco), 100 units/ml penicillin (Gibco), 100 *μ*g/ml streptomycin (Gibco), 10 *μ*M SB431542 (Wako), 100 nM LDN193189 (Wako), 20 ng/ml EGF (Peprotech), 20 ng/ml bFGF (Peprotech)) for next 6 days at 37 °C under humidified atmosphere of 5% CO_2_. Culture medium was changed every other day. Culture medium was finally switched to Neuron induction medium (Neurobasal medium (Gibco) supplemented with 1 × N2 (Gibco), 1 × B27 minus vitamin A (Gibco), 1 × GlutaMAX (Gibco), 100 units/ml penicillin (Gibco), 100 *μ*g/ml streptomycin (Gibco), 10 ng/ml BDNF (Peprotech), 10 ng/ml GDNF (Peprotech), 10 ng/ml NGF (Peprotech), 500 *μ*M dbcAMP (Sigma), 200 *μ*M ascorbic acid (Wako)) and was changed every other day for terminal differentiation. At 26 or 27 days of neuronal differentiation in total, neurons were used for further analyses.

Emergent NSCs were characterized according to the standard immunocytochemical protocol. Fluorophore-conjugated primary antibodies used were as follows: Cy3-conjugated anti-SOX2 (1:100 dilution; Millipore), AlexaFluor 488-conjugated anti-Nestin (1:100 dilution; Millipore). Stained samples were observed under a fluorescent microscope (IX71 System; Olympus).

Emergent neurons were characterized according to standard immunocytochemical protocol. Primary antibody used was anti-TUJ1 (1:200 dilution; Abcam). Secondary antibody used was AlexaFluor 568 (1:800 dilution; Molecular Probes). Stained samples were observed under a fluorescent microscope (IX71 System; Olympus).

### Analysis of mitochondrial respiration

Analysis of mitochondrial respiratory potential was performed using a flux analyzer (Seahorse XF^e^24 Extracellular Flux Analyzer; Seahorse Bioscience, North Billerica, MA, USA) with a Seahorse XF Cell Mito Stress Test Kit (Seahorse Bioscience) according to the manufacturer's instructions. Basal respiration and ATP production were calculated to evaluate mitochondrial respiratory function according to the manufacturer's instructions. After the measurement, cells were harvested to count the cell number, and each plotted value was normalized relative to the number of cells used. The detailed procedures are as follows:

For iPSCs, several pieces of iPSC colonies were transferred onto each well of Geltrex (Gibco)-coated XF^e^24 cell culture plates (Seahorse Bioscience) and were maintained in primate ESC culture medium. After 3 days in culture, iPSCs were equilibrated in unbuffered XF^e^ assay medium (Seahorse Bioscience) supplemented with 10 mM glucose, 1 mM sodium pyruvate and transferred to a non-CO_2_ incubator for 1 h before measurement. Oxygen consumption rate (OCR) was measured with sequential injections of 2 *μ*M oligomycin, 1 *μ*M FCCP and each 2 *μ*M of rotenone/antimycin A.

For iPSC-cardiomyocytes, each beating cardiomyocyte-aggregate at 23 days of differentiation in total was transferred onto each well of Geltrex (Gibco)-coated XF^e^24 cell culture plates (Seahorse Bioscience) and was maintained in Cardiac maturation medium. At 26 or 27 days of differentiation in total, beating cardiomyocytes were equilibrated in unbuffered XF^e^ assay medium (Seahorse Bioscience) supplemented with 10 mM glucose and 1 mM sodium pyruvate, and transferred to a non-CO_2_ incubator for 1 h before measurement. OCR was measured with sequential injections of 1 *μ*M oligomycin, 0.5 *μ*M FCCP and each 2 *μ*M of rotenone/antimycin A.

For iPSC-neurons, each neurosphere at 8 days of differentiation in total was transferred onto each well of Geltrex (Gibco)-coated XF^e^24 cell culture plates (Seahorse Bioscience) and was maintained in NSC expansion medium for next 6 days, followed by terminal differentiation in Neuron induction medium. At 26 or 27 days of differentiation in total, neurons were equilibrated in unbuffered XF^e^ assay medium (Seahorse Bioscience) supplemented with 10 mM glucose and 1 mM sodium pyruvate, and transferred to a non-CO_2_ incubator for 1 h before measurement. OCR was measured with sequential injections of 2 *μ*M oligomycin, 0.5 *μ*M FCCP and each 2 *μ*M of rotenone/antimycin A.

### Analysis of mitochondrial respiratory chain complex activity

Analysis of mitochondrial respiratory chain complex activity was performed according to our previous report:^20^ Briefly, mitochondrial respiratory complex activity was measured with Complex I Human Enzyme Activity Microplate Assay kit (Abcam) and with Complex IV Human Enzyme Activity Microplate Assay kit (Abcam) according to the manufacturer's instructions, respectively. Cell extracts (150 *μ*g for complex I, 50 *μ*g for complex IV) were used to measure time-dependent absorbance alterations on a multi-well plate reader (SPECTROstar Nano; BMG Labtech, Ortenberg, Germany).

### Neuroblastoma cell culture and neuronal differentiation

SH-SY5Y neuroblastoma cell line was maintained in Neuroblastoma growth medium (DMEM (Gibco) supplemented with 4.5 mg/ml glucose, 110 *μ*g/ml sodium pyruvate, 50 *μ*g/ml uridine (Sigma, St. Louis, MO, USA), 10% FBS (Gibco), 100 units/ml penicillin (Gibco), 100 *μ*g/ml streptomycin (Gibco)) at 37 °C under humidified atmosphere of 5% CO_2_. Culture medium was changed every other day.

For the establishment of mtDNA-depleted cell line (SH-SY5Y *ρ*^0^), sparsely plated SH-SY5Y cells were expanded in Neuroblastoma growth medium (DMEM (Gibco) supplemented with 4.5 mg/ml glucose, 110 *μ*g/ml sodium pyruvate, 50 *μ*g/ml uridine (Sigma), 10% FBS (Gibco), 100 units/ml penicillin (Gibco), 100 *μ*g/ml streptomycin (Gibco)) with the addition of 5 *μ*g/ml ethidium bromide to induce mtDNA depletion for at least 1 month in culture at 37 °C under humidified atmosphere of 5% CO_2_. Culture medium was changed every other day.

For neuronal lineage commitment, SH-SY5Y WT and SH-SY5Y *ρ*^0^ (1 × 10^5^ cells, respectively) were transferred onto Geltrex (Gibco)-coated multi-well culture plates or culture dishes and were maintained in Neuroblastoma growth medium (DMEM (Gibco) supplemented with 4.5 mg/ml glucose, 110 *μ*g/ml sodium pyruvate, 50 *μ*g/ml uridine (Sigma), 10% FBS (Gibco), 100 units/ml penicillin (Gibco), 100 *μ*g/ml streptomycin (Gibco)) at 37 °C under humidified atmosphere of 5% CO_2_. The next day, culture medium was replaced with Neuron induction medium (Neurobasal medium (Gibco) supplemented with 1 × N2 (Gibco), 1 × B27 minus vitamin A (Gibco), 1 × GlutaMAX (Gibco), 100 units/ml penicillin (Gibco), 100 *μ*g/ml streptomycin (Gibco), 10 ng/ml BDNF (Peprotech), 10 ng/ml GDNF (Peprotech), 10 ng/ml NGF (Peprotech), 500 *μ*M dbcAMP (Sigma), 200 *μ*M ascorbic acid (Wako)) with the addition of 50 *μ*g/ml uridine (Sigma) and was changed every other day for terminal differentiation. At 14 days of neuronal differentiation, neurons were used for further analyses. Time-lapse images of neuronal lineage commitment from day 4 to day 8 were also obtained using a live cell imaging system (BioStudio; Nikon Engineering) at 30 min of interval.

Emergent neurons were characterized according to standard immunocytochemical protocol. Primary antibody used was as follows: anti-NF-H (1:200 dilution; Abcam). Secondary antibody used was as follows: AlexaFluor 568 (1:800 dilution; Molecular Probes). Stained samples were observed under a fluorescent microscope (IX71 System; Olympus).

### Cytochemical COX staining

Cytochemical COX staining was performed as follows: Briefly, undifferentiated and differentiated SH-SY5Y WT and SH-SY5Y *ρ*^0^ were stained with COX reaction buffer (pH 5.5; 100 mM sodium acetate, 0.1% MnCl_2_, 0.001% H_2_O_2_, 10 mM diaminobenzidine) at 37 °C for 1 h, followed by subsequent incubation with 1% CuSO_4_ at 37 °C for 5 min. Stained samples were observed under a microscope (IX71 System; Olympus).
