# Supplementary material for: Mitochondrial respiratory dysfunction disturbs neuronal and cardiac lineage commitment of human iPSCs
Source: Cell Death Dis. 2017 Jan 12;8(1):e2551–. doi: 10.1038/cddis.2016.484 (PMC5386384; doi:10.1038/cddis.2016.484)
Supplement: Supplementary Information [file cddis2016484x6.doc]

**Supplementary information**

**Mitochondrial respiratory dysfunction disturbs neuronal and cardiac lineage-commitment of human iPSCs**

Short Title: **m.3243A>G impairs neuronal and cardiac maturation**

Mutsumi Yokota^1,3^, Hideyuki Hatakeyama^1,3,*^, Yasuha Ono^1^, Miyuki Kanazawa^2^, Yu-ichi Goto^1,2,3,*^

1. Department of Mental Retardation and Birth Defect Research, National Institute of Neuroscience, National Center of Neurology and Psychiatry, Tokyo 187-8502, Japan
2. Medical Genome Center, National Center of Neurology and Psychiatry, Tokyo 187-8551, Japan
3. AMED-CREST, Japan Agency for Medical Research and Development, Tokyo 100-0004, Japan

^*^ Corresponding Author:

Hideyuki Hatakeyama, PhD (**E-mail:** hideyuki@ncnp.go.jp)

Yu-ichi Goto, MD, PhD (**E-mail:** goto@ncnp.go.jp)

Department of Mental Retardation and Birth Defect Research, National Institute of Neuroscience,

National Center of Neurology and Psychiatry (NCNP)

**Address:** 4-1-1 Ogawahigashi, Kodaira, Tokyo 187-8502, Japan

**Phone:** +81-42-346-1713; **Fax:** +81-42-346-1743


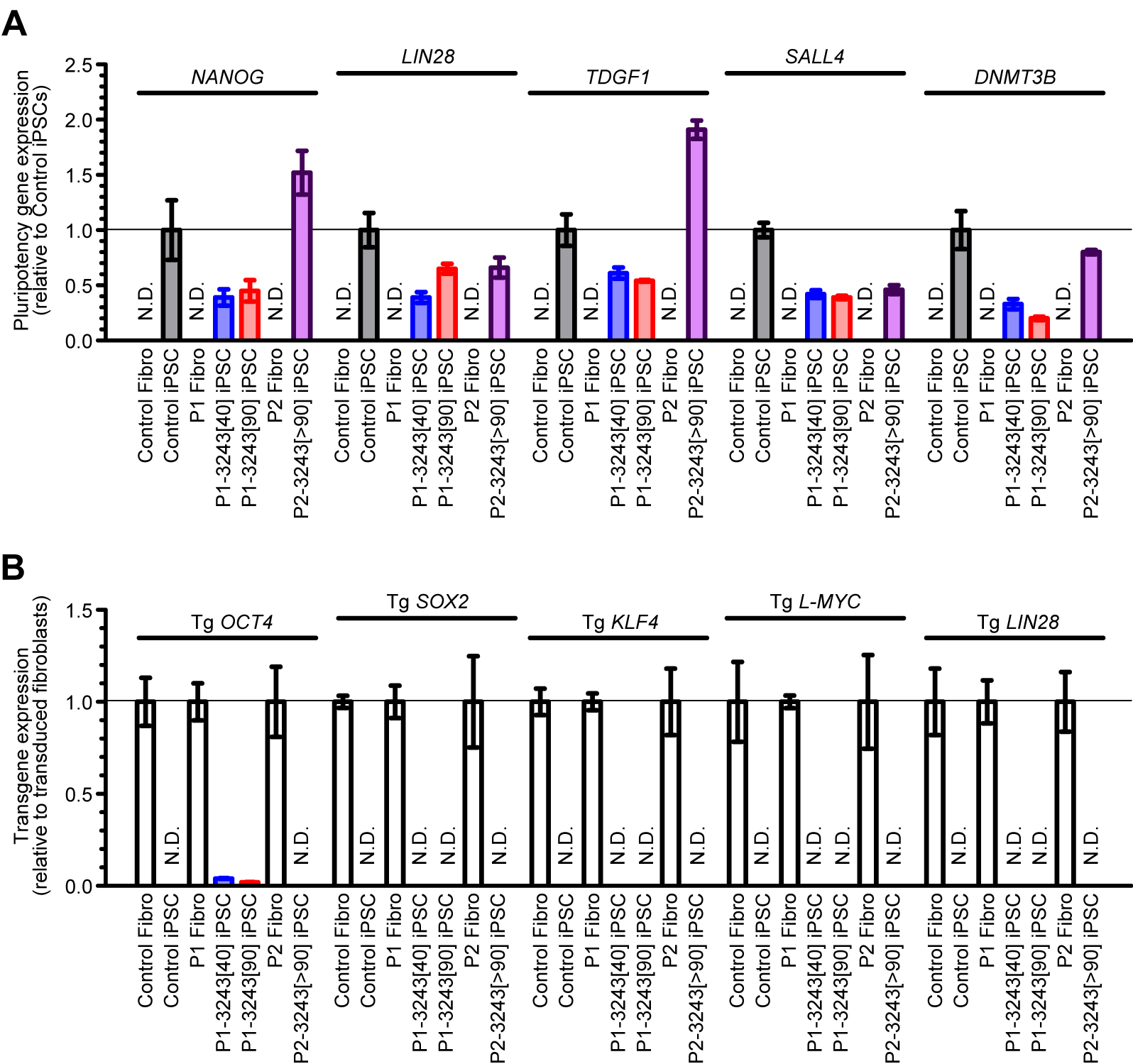


**Supplementary information, Figure S1. Analysis of pluripotency genes expression and transgenes silencing in all iPSC lines (related to Figure 1).**

**(A)** Expression of the representative pluripotency genes in all iPSC lines. Parental fibroblasts were also used as negative samples. Expression level of each pluripotency gene was calculated using ΔΔC_T_-based relative quantification method by real-time PCR. Measurements were performed in triplicate. N.D.: Not Detected.

**(B)** Silencing of transgenes expression in all iPSC lines. Transduced parental fibroblasts were also used as positive samples. Expression level of each transgene was calculated using ΔΔC_T_-based relative quantification method by real-time PCR. Measurements were performed in triplicate. N.D.: Not Detected.


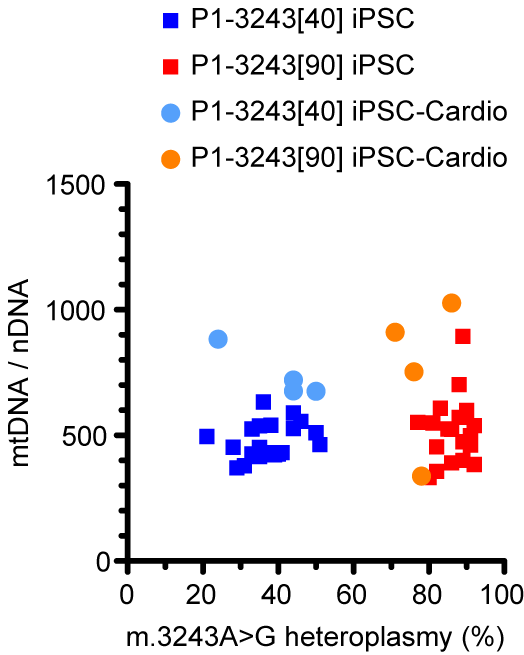


**Supplementary information, Figure S2. Relationship between the distributions of m.3243A>G proportions and mtDNA copy number per cell in iPSCs and iPSC-cardiomyocytes (related to Figure 2).**

No significant difference in mtDNA copy number was observed between P1-3243[40] and P1-3243[90] iPSC lines, or among their iPSC-cardiomyocytes.


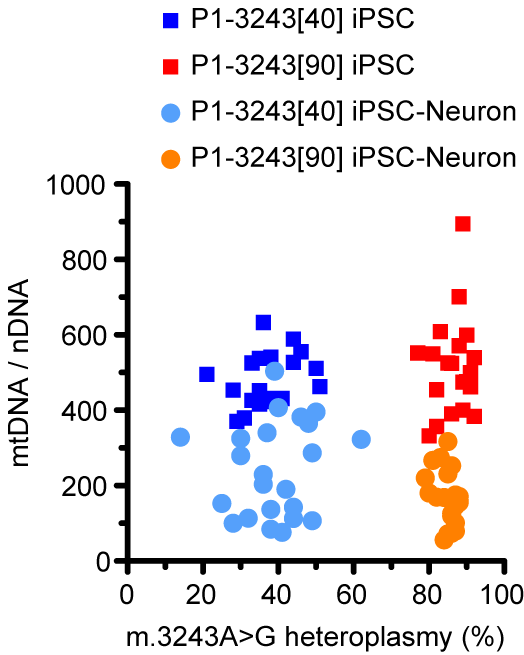


**Supplementary information, Figure S3. Relationship between the distributions of m.3243A>G proportions and mtDNA copy number per cell in iPSCs and iPSC-neurons (related to Figure 3).**

No significant difference in mtDNA copy number was observed between P1-3243[40] and P1-3243[90] iPSC lines, or among their iPSC-neurons.

**Supplementary information, Movie S1. Representative beating cardiomyocytes derived from control iPSC line (related to Figure 2).**

**Supplementary information, Movie S2. Representative beating cardiomyocytes derived from P1-3243[40] iPSC line (related to Figure 2).**

**Supplementary information, Movie S3. Representative beating cardiomyocytes derived from P1-3243[90] iPSC line (related to Figure 2).**

**Supplementary information, Movie S4. Time-lapse images of neuronal lineage-commitment in SH-SY5Y WT from day 4 to day 8 (related to Figure 4).**

**Supplementary information, Movie S5. Time-lapse images of neuronal lineage-commitment in SH-SY5Y ρ^0^ from day 4 to day 8 (related to Figure 4).**

**Supplementary information, Table S**1. Primer list.

| Primer | Forward | Reverse | Application |
| --- | --- | --- | --- |
| m.3243A>G_PCR | **[Biotin]**-CCCTGTACGAAAGGACAAGAGAAA | TGGGGCCTTTGCGTAGTTGTAT | m.3243A>G mutation analysis |
| m.3243A>G_PyroSeq |  | ATGCGATTACCGGGC | (Pyrosequencing) |
| *MT-CYB* (mtDNA) | TGCAACTATAGCAACAGCCTTCA | GAACTAGGTCTGTCCCAATGTATGG | mtDNA copy number |
| *GAPDH* (nDNA) | TTCAACAGCGACACCCACT | CCAGCCACATACCAGGAAAT |
| *NANOG* | CAGCCCCGATTCTTCCACCAGTCCC | CGGAAGATTCCCAGTCGGGTTCACC | Pluripotency genes expression |
| *LIN28* | TGCACCAGAGTAAGCTGCAC | CTCCTTTTGATCTGCGCTTC |  |
| *TDGF1* | CTGCTGCCTGAATGGGGGAACCTGC | GCCACGAGGTGCTCATCCATCACAAGG |  |
| *SALL4* | AGCACATCAACTCGGAGGAG | CCTGGGTGGTTCACTGGAG |  |
| *DNMT3B* | TGCTGCTCACAGGGCCCGATACTTC | TCCTTTCGAGCTCAGTGCACCACAAAAC |  |
| *Tg_OCT4* | CATTCAAACTGAGGTAAGGG | TAGCGTAAAAGGAGCAACATAG | Transgenes silencing |
| *Tg_SOX2* | TTCACATGTCCCAGCACTACCAGA | TTTGTTTGACAGGAGCGACAAT |  |
| *Tg_KLF4* | CCACCTCGCCTTACACATGAAGA | TAGCGTAAAAGGAGCAACATAG |  |
| *Tg_L-MYC* | GGCTGAGAAGAGGATGGCTAC | TTTGTTTGACAGGAGCGACAAT |  |
| *Tg_LIN28* | AGCCATATGGTAGCCTCATGTCCGC | TAGCGTAAAAGGAGCAACATAG |  |
| *PPIA* | GGTCCCAAAGACAGCAGAAAAT | ACCACCCTGACACATAAACCCT | Housekeeping gene |
| *TBP* | TTGCTGCGGTAATCATGAGG | TGGACTGTTCTTCACTCTTGGC |
